# Predicting Conservation Status of Testudoformes under Climate Change Using Habitat Models

**DOI:** 10.3390/ani14162300

**Published:** 2024-08-07

**Authors:** Wenbo Liao, Shun Cao, Ying Jiang, Weijie Shao, Li Zhao, Chengzhi Yan

**Affiliations:** 1 Key Laboratory of Southwest China Wildlife Resources Conservation (Ministry of Education), China West Normal University, Nanchong 637009, China; caoshun_0@163.com (S.C.); jiangyingjuly@163.com (Y.J.); somitsu@126.com (W.S.); lizhao_688@163.com (L.Z.); 2 Key Laboratory of Artificial Propagation and Utilization in Anurans of Nanchong City, China West Normal University, Nanchong 637009, China; 3 College of Panda, China West Normal University, Nanchong 637009, China; 4 School of Ecology and Nature Conservation, Beijing Forestry University, Beijing 100083, China

**Keywords:** conservation status, climate change, distribution range, extinction risk, Testudoformes

## Abstract

**Simple Summary:**

We investigated the effect of climate change on the potential distribution of 268 Testudines species and evaluated their future conservation status. Our analysis revealed that over half of these species are projected to experience a contraction in their distribution ranges due to climate change, leading to an increase in endangered species. In particular, three species currently classified as critically endangered—Three-striped roofed turtle (*Batagur dhongoka*), Durango mud turtle (*Kinosternon durangoense*), and Colombian mud turtle (*Kinosternon dunni*)—demonstrated over 80% potential distribution range reduction under future climate scenarios, resulting in the highest extinction risk. Additionally, our findings suggest that climate change may have positive effects on some Testudines species, with certain species potentially experiencing an expansion in their distribution ranges under future climate scenarios.

**Abstract:**

Climate change promotes variations in distribution ranges, potentially leading to biodiversity loss and increased extinction risks for species. It is crucial to investigate these variations under future climate change scenarios for effective biodiversity conservation. Here, we studied the future distribution ranges of 268 Testudoformes species under climate change using habitat models, specifically species distribution models (SDMs), to assess their conservation status. Our results have indicated that over half of species are projected to experience declines in their potential distribution ranges under two scenarios. In particular, we found that three critically endangered species—Three-striped roofed turtle (*Batagur dhongoka*), Durango mud turtle (*Kinosternon durangoense*), and Colombian mud turtle (*Kinosternon dunni*)—displayed extraction of their distribution ranges and faced extinction under global climate change. Additionally, our analysis revealed that the potential distribution ranges of some species might increase under future climate scenarios. However, these findings must be interpreted with caution as they do not account for other significant factors such as biological invasions, population structure, land-use change, anthropogenic disturbances, and inter-organism interrelationships. Future studies should incorporate these factors to provide a more comprehensive assessment of extinction risks. Our findings suggest that climate change, in conjunction with habitat degradation and human activities, must be considered when assessing the extinction risks of Testudoformes.

## 1. Introduction

Global climate change affects the abiotic–biotic interactions, leading to variations in species diversity, morphological traits, resource competition, physiological activity, genetic diversity, micro-habitat use, and distributional ranges in organisms [1,2,3,4,5,6,7,8,9,10,11,12,13,14,15,16,17,18,19,20,21,22]. Over 500 terrestrial vertebrates have been identified as facing extinction risk in the last 500 years [13,23,24]. The extinction risk for most species is primarily affected by habitat loss, biological invasions, overexploitation, environmental pollution, anthropogenic activity, toxification, and climate disruption [11,23,25,26]. While human-induced habitat destruction is widely recognized as a key factor driving biodiversity loss, climate change driven by anthropogenic activity is increasingly seen as a significant contributing factor, especially in the coming century [27,28]. Species often struggle to adapt to extreme climatic events (e.g., droughts, floods, and high temperatures) resulting from anthropogenic climate change, leading to habitat loss and posing significant threats to species survival [29]. Additionally, global climate changes are known to affect species’ ranges and migration patterns [30]. For instance, warming environmental temperatures are likely to trigger migration to higher latitudes and/or altitudes, forcing species to face formidable challenges in adapting to specific environments, thus exacerbating extinction risk due to reduced adaptive space [17].

There is substantial evidence that climate changes driven by anthropogenic activity affect potential distribution ranges and population variations, resulting in worldwide biodiversity loss [18,19,21]. The primary influence of global climate change on animals is the reduction or loss of suitable habitats, directly leading to species decline and potential extinction [15,27]. For instance, over 20% of 48 species of lizards in Mexico are projected to be at risk of extinction by 2080 due to climate change [3]. A study on the conservation status of 5282 reptile species under global climate change revealed that over 52.1% display a decrease in their distributional ranges, resulting in a rapid increase in the number of threatened species [29]. However, global climate changes have varying effects on the distribution patterns of different species. While some species are threatened, others continue to expand their distribution ranges [24]. Therefore, it is crucial to identify the most vulnerable species and areas by assessing the potential effects of global climate change on species distributions [31,32,33,34]. This targeted approach will help prioritize conservation efforts and mitigate the impacts of climate change on biodiversity.

Ectothermic vertebrates, such as reptiles, are highly vulnerable to global climate change due to their limited dispersal capabilities and climate-dependent physiological functions [35,36,37]. Reptiles are experiencing rapid population declines and increasing extinction rates, with over 21% of species listed as threatened due to global climate change [38,39,40,41]. For instance, population density declines in Costa Rican tropical rainforests since 1970 have been linked to climate-driven reductions in the quantity of standing leaf litter [38]. Climate changes (e.g., variations in rainfall and temperature) are significant factors affecting the distribution and conservation status of reptiles [39,40,41,42,43]. Hence, climate change will affect the potential distribution ranges of reptiles, thereby impacting their biodiversity. In contrast, a recent study has revealed that over 500 species of reptiles are experiencing a decrease in potential distribution range and facing extinction risk over the current century [37].

Testudoformes, a group of highly specialized reptiles, are distinguished by their unique adaptations for life on land. This group encompasses a diverse range of species with varying dietary habits: many are herbivorous, consuming a variety of plant materials; some are carnivorous, feeding on invertebrates and small vertebrates; and others are omnivorous, incorporating both plant and animal matter into their diets [44,45]. These turtles are characterized by their rigid shells, which provide protection from predators, and their ability to retract their heads and limbs into their shells. Primarily found in a variety of terrestrial habitats, including forests, grasslands, and deserts, Testudoformes lay eggs on land, with females selecting suitable nesting sites to ensure the successful development of their offspring [45]. During the breeding season, males often display brighter colors as part of their mating behavior. Most Chelonian species, including Testudoformes, are experiencing significant declines in population density and potential distribution ranges due to anthropogenic activities such as habitat destruction, pollution, and climate change [46]. Climate change, in particular, poses a severe threat by altering temperature and precipitation patterns, which can affect the availability of suitable habitats and the physiological processes of these ectothermic animals [31,36,37].

The primary aim of this study is to assess the future conservation status of Testudoformes by predicting the potential distribution ranges for 268 global species under current (using baseline data from 1970 to 2000) and future climate scenarios (2040–2060 and 2060–2080) using the habitat model. This assessment will provide insights into how climate change may impact these species and help identify those at the highest risk of extinction. We hypothesize that climate change will lead to significant contractions in the potential distribution ranges of most Testudoformes species, increasing their extinction risk. Conversely, we expect that some species may experience range expansions, particularly in higher latitudes, due to changing climatic conditions.

## 2. Materials and Methods

### 2.1. Species Distribution Models

MaxEnt model, an essential tool for predicting species distribution, is known for its accuracy in forecasting and its ability to project across space and time [47]. Utilized extensively to assess the conservation status of endangered species in the face of climate change, species distribution models (SDMs) can anticipate where species may inhabit under various environmental scenarios [48,49,50,51]. There is evidence that the MaxEnt model has advantages for robustness with limited data points, low sensitivity to collinearity among variables, and the capacity to model intricate associations among variables [52,53]. As a result, it is a widespread application in conservation biology for exploring species’ ecological niche shifts, assessing habitat suitability, and discussing the influences of climate change on the conservation status of specific species [54,55,56]. The utilization of the MaxEnt model to forecast the potential distribution ranges of Testudoformes in the future, considering a range of 1 to 1000 occurrence records, is a logical approach in this context.

Modeling was conducted solely using presence data with MAXENT Version 3.4.3 to address the challenge of limited occurrence records for certain species [57,58,59]. For each species, 75% of occurrence points were randomly chosen as training data for model construction, with the remaining 25% allocated for validation. Assessment of model weights was carried out using the Jackknife method, and 10 iterations of Receiver Operating Characteristic (ROC) curves were generated. The background points were limited to a maximum of 100,000, with the Logistic output type selected. The predictive accuracy of the model was evaluated using the Area Under Curve (AUC) of ROC curves [60]. The average results from 7 models were transformed into a raster format using the transformation tool in ArcGIS 10.8 [57]. Jenks’ natural breaks method was employed to classify the results into four categories: high-habitability, medium-habitability, low-habitability, and non-habitability zones. The potential distribution range of species was determined by combining the first three categories [61,62].

### 2.2. Occurrence Records

We obtained occurrence records of Testudoformes from online databases such as the Global Biodiversity Information Facility (GBIF) (https://www.gbif.org/, accessed on 15 December 2022) and the Reptile Database (http://www.reptile-database.org/, accessed on 15 December 2022), as well as data published in the primary scientific literature [31,37]. Data, including precise geographical coordinates from GBIF, were initially compiled covering the period from 1970 to 2023 (accessed in May 2023). In addition, we collected intensive data from the literature to supplement the global occurrence records. Our dataset comprised data for 268 Testudoformes species, totaling 195,358 occurrence records. These records included 132 species distributed in North America, 39 in Oceania, 56 in Africa, 49 in South America, 43 in Europe, and 103 in Asia. There were no species recorded in Antarctica, as Testudoformes do not inhabit this region. This dataset covers 75.1% (268 out of 357) of known Testudoformes species to date (Table S1), with 24.9% of species not displaying occurrence points (Table S2). We did not include some rare and small-ranged species due to the scarcity of available occurrence records, which limits the accuracy of species distribution models (SDMs). Species with fewer than 4 occurrence records (Table S3) were excluded from our analyses to ensure the reliability of the predictions, as low sample sizes can limit SDM accuracy [55]. Consequently, we simulated the potential distribution areas of 268 species with four or more records.

### 2.3. Environmental Predictors

A total of 19 bioclimatic variables, which included current (1970–2000) and two future (2050s [2041–2060] and 2070s [2061–2080]) scenarios at a spatial resolution of 10 min (approximately 18.5 km at the equator), were obtained from the WorldClim database (https://www.worldclim.org/, accessed on 15 December 2022). For future scenarios, we provided the bioclimatic data for the 2050s and 2070s based on the mean values from 2041 to 2060 and 2061 to 2080, respectively. The 2050s and 2070s are critical for long-term planning and policy-making. Assessing the potential impacts of climate change on biodiversity over these periods provides valuable insights for developing effective conservation strategies and policies. These timeframes are particularly relevant for international agreements and commitments, such as those under the United Nations Framework Convention on Climate Change (UNFCCC). To project future climate change, we employed general circulation models (GCMs) associated with Shared Socio-economic Pathways (SSPs) from the Coupled Model Intercomparison Project Phase 6 (CMIP6) by the IPCC [51]. The BCC-CSM2-MR climate system model from the Beijing Climate Center was utilized especially [52]. Two scenarios, SSP2-4.5 and SSP5-8.5, were selected. SSP2-4.5 envisions slower population growth by mid-century, balanced energy consumption, moderate greenhouse gas emissions, moderate economic advancement, and technological progress based on moderate climate policies [63]. SSP5-8.5 represents rapid population growth until the century’s end, increased energy use, the continued rise in greenhouse gas emissions, rapid economic growth, and technological innovation without strong climate policy [63].

Multicollinearity among the climatic variables was addressed using Pearson correlation analysis, with the exclusion of variables showing correlations greater than 0.8. Subsequently, the ‘vif’ function in the ‘usdm’ R package was utilized to calculate the variance inflation factor (VIF) for the remaining variables, and VIF values exceeding 10 were excluded from the initial bioclimatic variables for each species [54,55]. In total, we retained 10 climate variables to construct species distribution models. Ten climate variables were retained for constructing species distribution models, including BIO2, BIO4, BIO5, BIO8, BIO9, BIO12, BIO14, BIO15, BIO18, and BIO19, representing aspects like diurnal range, temperature variability, precipitation, and seasonality. Altitude data were acquired from Earthdata (https://www.earthdata.nasa.gov/, accessed on 15 December 2022).

### 2.4. Endangerment Estimate

Variations in species richness are primarily driven by changes in species distribution ranges. To analyze the shifting distribution ranges of Testudoformes under current and future scenarios, we mapped species richness for each one-degree cell. By comparing species richness between the present and future scenarios, we calculated variations in species richness within each cell, which ultimately determined the influences of climate change on the distribution range change of Testudoformes.

The adjustments in the IUCN rankings of endangered species primarily hinge on the pace of distribution range changes within Testudoformes. The future endangerment estimates of Testudoformes referred to the IUCN Endangered Species Criteria A3(C) [58,59,60]. Our classification criteria categorize extinct (EX) species as those losing 100% of their potential range, Critically Endangered (CR) species losing 80%, Endangered (EN) species losing 50%, Vulnerable (VU) species losing 30%, Near Threatened (NT) species losing 10%, and the remaining as Least Concern (LC).

As our model data originate from occurrence records, species with limited occurrence points may reflect sampling challenges or small population sizes during fieldwork. With biodiversity facing a decline under global climate change, most species are facing significant threats. When the simulation of potential distribution areas was not observed in some species, we calculated the area enclosed by these points for Testudoformes with three occurrence points. The endangerment status is elevated by three levels for potential distribution areas below 100 square kilometers, by two levels below 500 square kilometers, and by one level below 10,000 square kilometers. When comparing the distance between two records within Testudoformes, an endangerment rating increase is three levels below 10 km, two levels below 50 km, and one level below 500 km. Species in Testudoformes have only one record; the endangerment risk is elevated by three levels. Some species display limited sample sites; we assume that the endangered status of these species will not change between the 2050s and 2070s.

## 3. Results

**Figure 1:**
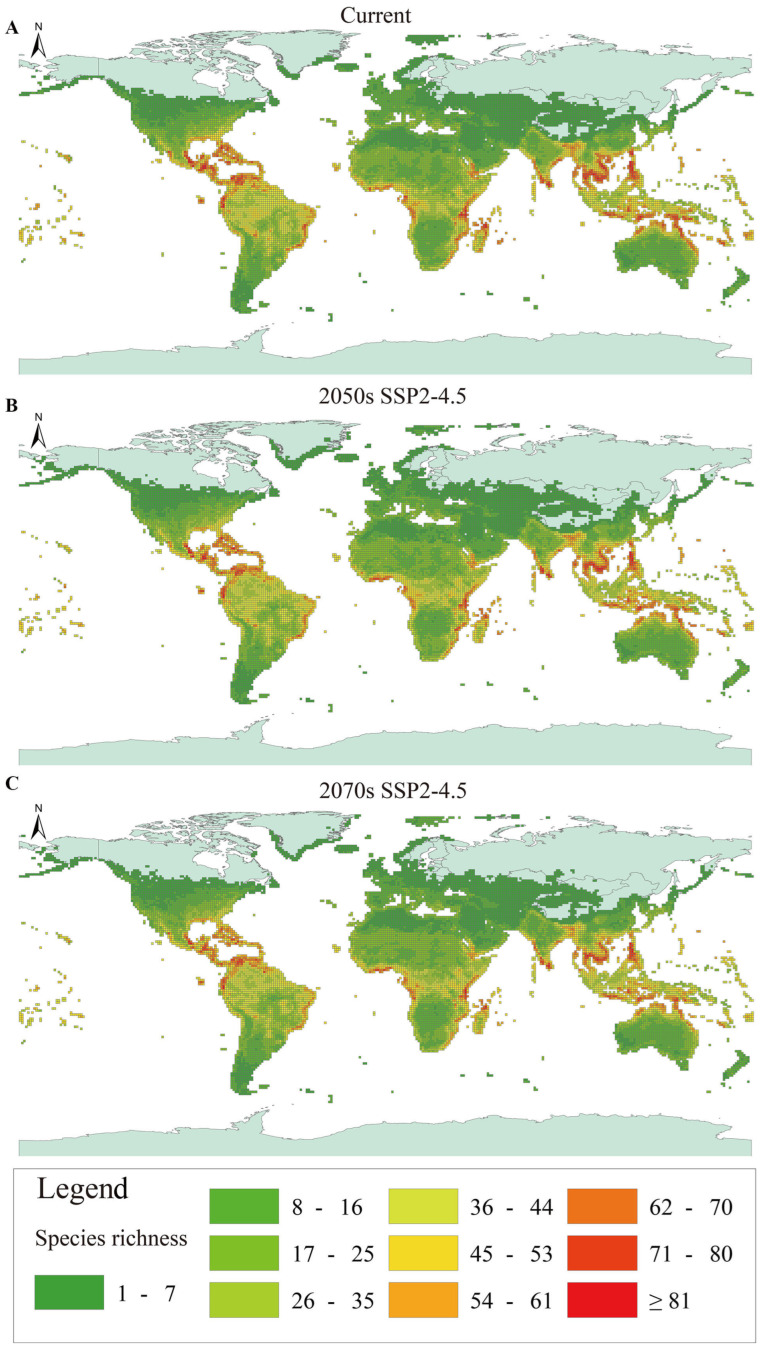
(**A**) Current spatial pattern for species richness. (**B**,**C**) Projected spatial pattern for species richness under the SSP2-4.5 scenario.

The MaxEnt model displayed excellent predictive performance for all analyzed species (AUC train = 0.99, AUC test = 0.99, 2050s; AUC train = 0.99, AUC test = 0.99, 2070s). Hence, the MaxEnt model keeps high accuracy and reliability in predicting the distribution range of Testudoformes under climate change (Figure 1). We found varying trends in the species richness of Testudoformes under climate change, with some regions experiencing a decrease in species richness while others show an increase. Specifically, South America, the central and eastern coast of Africa, and Southeast Asia are experiencing a high amount of habitat loss, with China and other Asian regions being the least affected (Figure 1, Table 1). This suggests that climate change has a complex and region-specific impact on Testudoformes species distribution. Therefore, the qualitative effect of climate change on Testudoformes is not uniform and varies significantly depending on geographic location and individual species’ adaptability.

To provide a more detailed overview of the situation in China, we have included additional explanatory materials and photographs of model species that represent different trends (Figure S1). For instance, the Snapping turtle (*Chelydra serpentina*) is one of the most climate-affected chelonian species in the region, with its potential distribution in China nearly disappearing due to global climate change. In contrast, the Chinese three-striped box turtle (*Cuora trifasciata*) is one of the species less affected by climate-induced habitat loss in China. Furthermore, species such as the Indochinese box turtle (*Cuora galbinifrons*) and the Black-breasted leaf turtle (*Geoemyda spengleri*) illustrate that their distribution ranges may remain stable or even expand under future climate scenarios.

**Figure 2:**
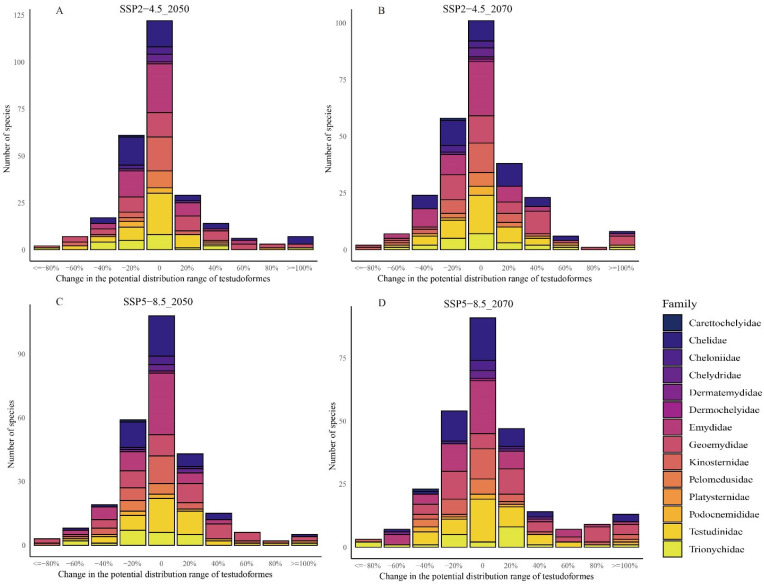
Proportional alteration in distribution range for IUCN Red List species in the years 2050 and 2070 predicted by MaxEnt projections. (**A**,**B**) Projected changes under the SSP2-4.5 scenario. (**C**,**D**) Projected changes under the SSP5-8.5 scenario. Negative values on the x-axis indicate a loss of suitable habitat.

The MaxEnt model revealed that over 48.51% of species for Testudoformes will possibly experience a decrease in potential distribution ranges from the present time to 2050s and 2070s under SSP2-4.5 and SSP5-8.5 scenarios (Figure 2). To provide detailed information, we have included tables (Table S4; Table S5) listing the genera and families of the Testudoformes species projected to experience these distribution changes. We found that 58.21% (2050s) and 54.48% (2070s) of species display a decreased trend in potential ranges under the SSP2-4.5 scenario. To provide clarity, we have included detailed information on the specific species affected (Table S4; Table S5). For instance, species such as *Astrochelys radiata* (Radiated Tortoise), *Graptemys pearlensis* (Pearl River map turtle), and *Cyclemys_dentata* (Asian leaf turtle) are among those projected to experience significant reductions in their potential distribution ranges. Under the SSP5-8.5 scenario, 52.61% (2050s) and 48.51% (2070s) of species were projected to have reduced their distribution ranges. Over 80% of the potential distribution ranges of the two species (*Batagur dhongoka* and *Kinosternon dunni*) were projected to decline (2070s, SSP2-4.5), while it was projected to decrease for *B. dhongoka* and *K. durangoense* from the present time to 2050s and 2070s under the SSP5-8.5 scenario, which was regarded as the critically endangered species. These projected declines are primarily due to both *B. dhongoka* and *K. durangoense* depending on specific habitats such as rivers and wetlands, which are vulnerable to climate change-induced alterations in temperature and precipitation patterns, leading to habitat degradation and fragmentation. The three critically endangered species displayed extraction of their distribution ranges and faced the highest extinction risk under global climate change. By specifically describing the changes in distribution for these critically endangered and endangered species, we aim to underscore the immediate need for targeted conservation efforts to mitigate the impacts of climate change on these vulnerable species.

We also found that the potential distributions ranging from 50% to 80% of nine (2050s, SSP2-4.5) and seven (2070s, SSP2-4.5) species and ten (2050s, SSP5-8.5) and nine (2070s, SSP5-8.5) species were projected to decline and regarded as the endangered species.

**Figure 3:**
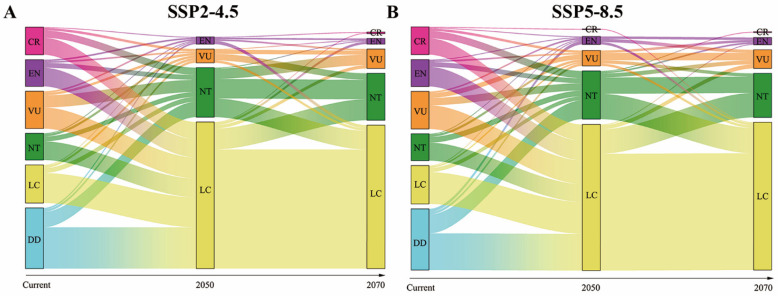
(**A**,**B**) Alterations of IUCN Red List categories for Testudoformes across time under SSP2-4.5 and SSP5-8.5 scenarios, respectively. The x-axis denotes the temporal scale, with the current status per the IUCN and projections for 2050 and 2070 based on distribution range modifications. The line’s thickness correlates with the species count. Data Deficient (DD); Least Concern (LC); Near Threatened (NT); Vulnerable (VU); Endangered (EN); Critically Endangered (CR); Extinct (EX).

The MaxEnt model showed that 17 species and 24 species would lose currently suitable habitats ranging from 30% to 50% from the current time to 2050s and 2070s under the SSP2-4.5, while 19 and 23 species were observed in the SSP5-8.5 scenarios, which became the vulnerable species. There are 61 (2050s, SSP2-4.5) and 59 (2050s, SSP5-8.5) species and 58 (2070s, SSP2-4.5) and 54 (2070s, SSP5-8.5) species whose current suitable habitats ranging from 10% to 30% were projected to decline, which became near-threatened species. Finally, the potential distribution ranges of 69 (2050s, SSP2-4.5) and 55 (2070s, SSP2-4.5) species and 54 (2050s, SSP5-8.5) and 43 (2050s, SSP5-8.5) species were projected to decline less than 10%, which were regarded as least concern species. In total, the number of all Testudoformes for threat was projected to decrease by 6.99% in 2050s and 70.79% in 2070s under the SSP2-4.5 scenario, while it was projected to decrease by 73.68% in 2050s and 71.05% in 2070s under the SSP5-8.5 scenario. In particular, the number of near-threatened species was projected to increase by 84.85% (2050s, SSP2-4.5) and by 75.76% (2070s, SSP2-4.5), or 78.79% (2050s, SSP5-8.5) and 63.64% (2070s, SSP5-8.5) (Figure 3).

The MaxEnt model also predicted an expansion of the potential distribution ranges of Testudoformes under future climate change. We found that 21 and 20 critically endangered species of Testudoformes displayed an increase in the potential distribution ranges in the 2050s under SSP2-4.5 and SSP5-8.5 scenarios, respectively, which were classified as least concern species. We also found that the potential distribution ranges of 22 and 23 endangered species of Testudoformes were projected to increase in the 2070s under SSP2-4.5 and SSP5-8.5 scenarios, respectively, and also classified as least concern species.

## 4. Discussion

We indicate significant shifts in the distribution patterns of Testudoformes in response to global climate change, with more than 48.51% of species showing a contraction in their distribution ranges under the SSP2-4.5 and SSP5-8.5 scenarios. Especially, over 80% of the potential distribution range of three species currently classified as critically endangered—Three-striped roofed turtle (*Batagur dhongoka*), Durango mud turtle (*Kinosternon durangoense*), and Colombian mud turtle (*Kinosternon dunni*)—face reduction under future climate scenarios, resulting in the highest extinction risk. Furthermore, certain species of Testudoformes are projected to experience expanded potential ranges as a result of climate change. Specifically, more than 21 Endangered (EN) species have transitioned to Least Concern (LC) status. This study provides valuable insights into the effects of future climate change on the potential distribution of Testudoformes and highlights the influence of habitat degradation and human activities on Testudoformes’ biodiversity in the context of global climate change. We suggest a notable increase in habitat range for the population growth of three species currently classified as critically endangered and make some conservation measures to decline the effect of human activity on habitats.

Previous studies have revealed substantial morphological, ecological, geographic, and conservation-related variations in wildlife in response to global climate change [64,65,66,67,68,69,70,71]. The impact of climate change on species distributions introduces a complex and context-dependent interplay of advantages and disadvantages. Environmental variables such as temperature and precipitation, linked to climate change, drive modifications in wildlife habitats, prompting migration to more conducive climatic zones to enhance survival prospects [68,72]. Many Testudoformes have temperature-dependent sex determination (TSD), where the sex of hatchlings is determined by the incubation temperature of the eggs. This phenomenon significantly impacts sex ratios, as rising temperatures could skew the ratio towards one sex, potentially leading to long-term population imbalances and affecting the genetic diversity and viability of populations [66]. Mounting evidence indicates that climate change directly influences fluctuations in environmental temperature and precipitation, indirectly leading to the contraction of wildlife distribution ranges [73]. Our findings suggested that over 48.51% of Testudoformes species displayed decreased trends in potential distribution ranges due to climate change projections up to the 2050s and 2070s. By contrast, the dispersal abilities of some species affect the extent of distribution range in response to climate change. Species with robust dispersal abilities may expand their distribution ranges, provided their tolerance thresholds adjust to climate change-induced shifts. [24]. For instance, many species can expand the distribution boundaries and increase space for survival and reproduction by adapting to environmental changes within new ranges and adjusting to new interspecific dynamics in the face of climate warming [74]. Our study successfully predicted an increase in the potential distribution ranges for some critically endangered species of Testudoformes, which were classified as least concern species under future climate conditions.

Understanding changes in habitat utilization by animals under climate change is paramount as it offers insights into shifts in their distribution ranges [74]. Studies on the effects of climate change on habitat use in various animal groups have shown a significant contraction of distribution ranges in projected future climatic conditions. For example, habitat suitability of alpine birds is predicted to decrease by 36.83% to 60.10% [75]. Furthermore, a substantial contraction in distribution ranges affecting over 79% of mountain vipers in the eastern Mediterranean poses an elevated risk of extinction for this species group [76]. Similar studies on the effects of climate warming on the reduction of distribution ranges in amphibians in the Near and Middle East have corroborated these findings [77,78]. Our study demonstrates a marked reduction in habitat ranges for a majority of species under climate change, contributing to the overall decline in distribution ranges. Of particular concern are the consequences of human activities on endangered species, with anticipated reductions in distribution ranges under climate change exacerbating extinction risks [79,80]. Our findings suggest that some endangered species face increased extinction risks under future climate scenarios, indicating a contraction in their distribution ranges due to escalating human disturbances. In addition, the contraction of species’ distribution ranges is likely attributed to habitat fragmentation, impeding the dispersal of juvenile individuals [81,82,83,84]. The species identified in our study experiencing distribution contractions are susceptible to habitat fragmentation, necessitating the establishment of well-designed protected areas for endangered species of Testudoformes under changing climatic conditions.

Fluctuations in environmental conditions play a pivotal role in influencing the body temperature of poikilothermic animals, subsequently impacting their distribution ranges and survival [82,85]. Notably, environmental temperature variations are regarded as an important factor constraining the potential distribution of snake species, as excessively high temperatures often correlate with the declined population density [86]. In addition, the emissions of greenhouse gases are linked to global climate warming [87]. As a result, rising temperatures under future climate change will result in diminishing the distribution range and suitable habitat areas for reptiles [29,88]. Consistent with our findings, increased temperatures have been shown to induce a pronounced reduction in the potential distribution area of Testudoformes species in the 2050s and 2070s within the framework of future climate change projections. Nonetheless, due to our exclusion of dispersal capacity in the analysis, there is a possibility that we underestimated the potential expansions in species’ ranges in response to climate warming.

Numerous studies on changes in distribution ranges and conservation status in animals have shown a concerning trend whereby certain species exhibit diminishing distribution ranges coupled with an augmented number of endangered species [6,7,18,32]. For example, although the management department provides for future protected areas in Morocco, this region remains highly susceptible to climate change, and the distribution ranges for reptiles remain on a declining trend [89,90]. In our study, we found that over 52.1% of Testudoformes species will face an increased risk of extinction under climate change. Our investigations highlight a discernible contraction in distribution ranges for the majority of Testudoformes species, projecting a trajectory of decline from present conditions to the years 2060 and 2070, driven by escalating temperatures that diminish habitat suitability as a consequence of climate warming. Our study at first provided a comprehensive estimation of the influence of global climate change on the conservation status of Testudoformes species. Consequently, we advocate for the mitigation of human activities within conservation habitats, particularly prioritizing the protection of five endangered species that are exceptionally susceptible to extinction risks. In particular, the three species of turtles (*B. dhongoka*, *K. durangoense*, and *K. dunni*), which possess small distribution ranges at the present time, have been classified as critically endangered species. Over 80% of their distributions will still be projected to decline in the 2050s and 2070s, which is likely to lead to extinction in the future. As such, urgent conservation measures, such as artificial breeding programs and habitat preservation, are recommended to safeguard their suitable habitats and ensure the preservation of viable populations.

Under the influence of global climate change, certain species adopt migration and dispersal as strategies to affect distribution ranges and conservation status. For instance, species may extend their distribution ranges in response to boundary shifts facilitated by their high dispersal capabilities [24]. In this study, we found a projected increase in the potential distribution ranges of three critically endangered Testudoformes species, indicating their robust adaptive capacity to future climate change scenarios. We inferred that these endangered species of Testudoformes were likely to exhibit heightened migration and dispersal behaviors, leading to significant improvements in their adaptive responses to anticipated climate change impacts.

The assessment of species endangerment heavily depended on evaluating changes in potential distributional ranges [31,91,92,93,94,95]. However, achieving an accurate estimation of extinction risk in Testudoformes necessitates considering the collective effects of biological invasions, population structure, land-use change, anthropogenic disturbances, and inter-organism interrelationships. Our study focused primarily on the potential distributional changes due to climate change, which is a critical factor but not the sole determinant of extinction risk. Future studies should incorporate these additional factors into analyses to enhance our comprehension of the endangerment status in Testudoformes with greater accuracy. Moreover, limitations such as the availability of occurrence records, the precision of climate models, and the exclusion of specific ecological interactions should be acknowledged. Addressing these limitations will provide a more holistic understanding of the threats faced by Testudoformes.

## 5. Conclusions

Our study highlights the significant impact of climate change on the potential distribution of Testudoformes, revealing a marked contraction in their ranges from the present to the 2050s and 2070s. Notably, species such as *B. dhongoka* (Dhond roofed turtle) and *K. dunni* (Colombian mud Turtle) from the Testudinidae family are projected to face severe range reductions, increasing extinction risks, particularly in Madagascar and the Galápagos Islands. Conversely, some species like *Geoclemys hamiltonii* (Spotted-pond turtle) from the Geoemydidae family may expand their ranges in Southeast Asia due to favorable climatic shifts. Temperature and precipitation changes are the primary drivers, with human activities exacerbating habitat loss, especially in tropical regions. Future research should further explore these climatic effects and their interaction with anthropogenic pressures to better inform conservation strategies.

## Figures and Tables

**Table 1:** Projected species richness of Testudoformes across continents under current and future climate scenarios.

Continent	Current	SSP2-4.5		SSP5-8.5	
		2050s	2070s	2050s	2070s
North America	132	134	133	133	132
South America	49	49	50	51	51
Asia	103	102	102	101	104
European	43	42	41	38	42
Oceania	39	39	40	41	41
Africa	56	53	52	57	57

## Data Availability

The data presented in this study are available on request from the corresponding author.

## Supplementary Materials

The following supporting information can be downloaded at: https://www.mdpi.com/article/10.3390/ani14162300/s1. Figure S1: Projected spatial pattern for species richness under the SSP5-8.5 scenario for the periods 2040–2060 and 2050–2070; Figure S2: (A) *Chelydra serpentina*, (B) *Cuora trifasciata*, (E) and (C) *Cuora galbinifrons*, (D) *Geoemyda spengleri* (International Union for the Conservation of Nature. The IUCN List of Threatened Species. [DB/OL]. https://www.iucnredlist.org/. 2024-01; Table S1: List of 268 Testudoformes species; Table S2: Species without occurrence points; Table S3: Species with fewer than four occurrence points; Table S4: Species with decreased distribution ranges under future climate scenarios; Table S5: Species with expanded distribution ranges under future climate scenarios.
