# Supplementary figures and images for: Predicting Conservation Status of Testudoformes under Climate Change Using Habitat Models
Source: Animals (Basel). 2024 Aug 7;14(16):2300. doi: 10.3390/ani14162300 (PMC11350788; doi:10.3390/ani14162300)

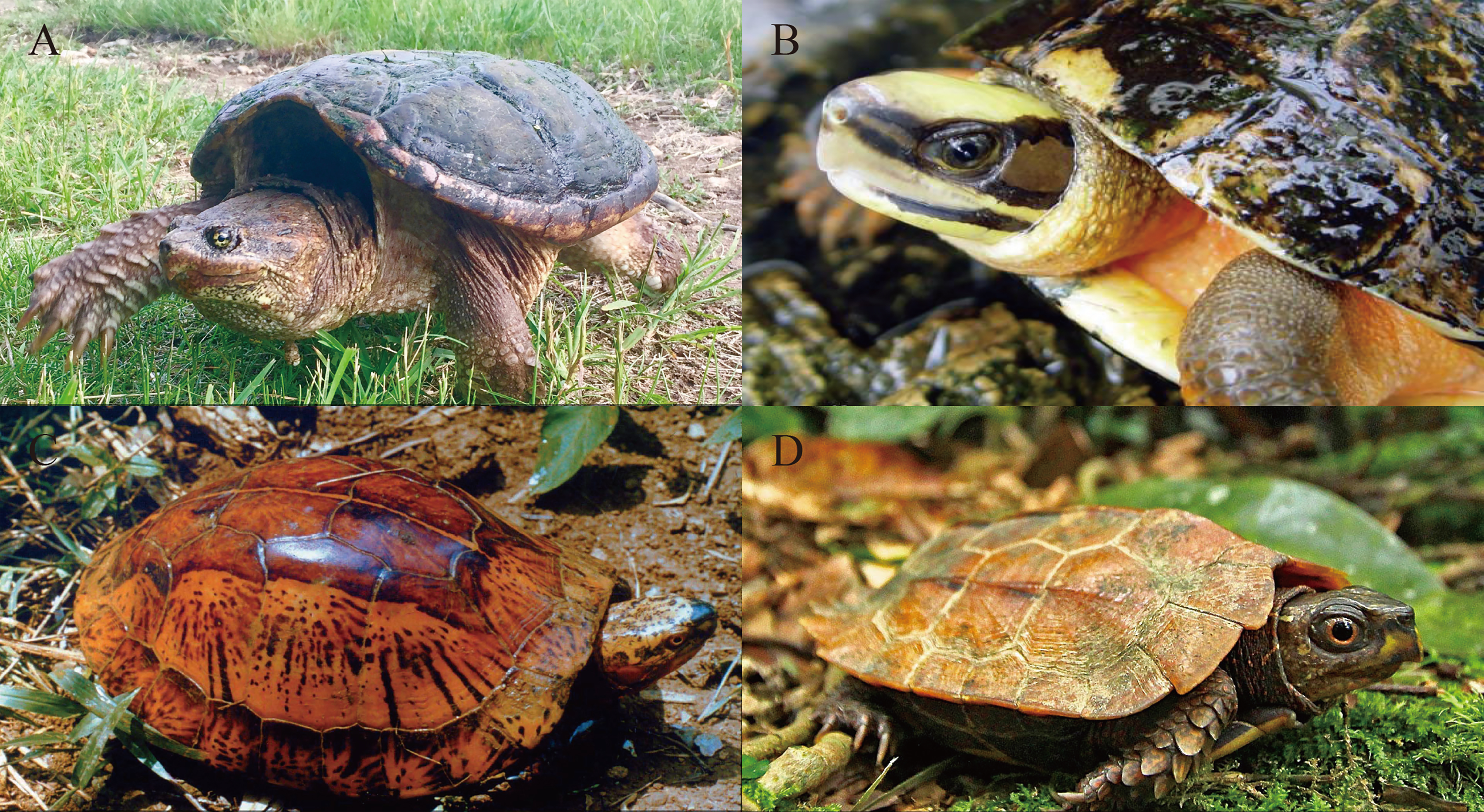

Supplement: Supplementary file 1 [file animals-14-02300-s001.zip › Figure S2.jpg]
